# Tumors Provoke Inflammation and Perineural Microlesions at Adjacent Peripheral Nerves

**DOI:** 10.3390/cells9020320

**Published:** 2020-01-29

**Authors:** Jennifer Cohnen, Lisa Kornstädt, Lisa Hahnefeld, Nerea Ferreiros, Sandra Pierre, Ulrike Koehl, Thomas Deller, Gerd Geisslinger, Klaus Scholich

**Affiliations:** 1 Institute of Clinical Pharmacology, University Hospital Goethe University Frankfurt, 60590 Frankfurt, Germany; jcohnen@outlook.de (J.C.); Kornstaedt@med.uni-frankfurt.de (L.K.); hahnefeld@med.uni-frankfurt.de (L.H.); ferreirosbouzas@em.uni-frankfurt.de (N.F.); spierre@em.uni-frankfurt.de (S.P.); geisslinger@em.uni-frankfurt.de (G.G.); 2 Fraunhofer Cluster of Excellence for Immune-Mediated Diseases (CIMD), 60596 Frankfurt/Main, Germany; ulrike.koehl@izi.fraunhofer.de; 3 Fraunhofer Institute for Cell Therapy and Immunology (IZI), 04103 Leipzig, Germany; 4 Institute of Clinical Immunology, University of Leipzig, 04103 Leipzig City, Germany; 5 Institute of Clinical Neuroanatomy, Dr. Senckenberg Anatomy, Neuroscience Center, Goethe-University Frankfurt, 60590 Frankfurt, Germany; t.deller@em.uni-frankfurt.de; 6 Fraunhofer Institute for Molecular Biology and Applied Ecology IME, Project Group Translational Medicine and Pharmacology, 60596 Frankfurt/Main, Germany

**Keywords:** dendritic cells, macrophage, microlesions, perineurium, sciatic nerve, tumor pain

## Abstract

Cancer-induced pain occurs frequently in patients when tumors or their metastases grow in the proximity of nerves. Although this cancer-induced pain states poses an important therapeutical problem, the underlying pathomechanisms are not understood. Here, we implanted adenocarcinoma, fibrosarcoma and melanoma tumor cells in proximity of the sciatic nerve. All three tumor types caused mechanical hypersensitivity, thermal hyposensitivity and neuronal damage. Surprisingly the onset of the hypersensitivity was independent of physical contact of the nerve with the tumors and did not depend on infiltration of cancer cells in the sciatic nerve. However, macrophages and dendritic cells appeared on the outside of the sciatic nerves with the onset of the hypersensitivity. At the same time point downregulation of perineural tight junction proteins was observed, which was later followed by the appearance of microlesions. Fitting to the changes in the epi-/perineurium, a dramatic decrease of triglycerides and acylcarnitines in the sciatic nerves as well as an altered localization and appearance of epineural adipocytes was seen. In summary, the data show an inflammation at the sciatic nerves as well as an increased perineural and epineural permeability. Thus, interventions aiming to suppress inflammatory processes at the sciatic nerve or preserving peri- and epineural integrity may present new approaches for the treatment of tumor-induced pain.

## 1. Introduction

The growth of primary tumors or metastases in the proximity of peripheral nerves occurs frequently, induces neuropathic pain in cancer patients, and is one of the major causes of intractable cancer pain [1,2]. Around 40% of cancer patients suffer from pain that includes a neuropathic component and around 30% the pain syndrome was directly related to tumor involvement of nervous tissue [1]. This cancer-induced neuropathic pain is generally believed to be based on mechanical nerve compression or tumor cell infiltration of the nerve. In these patients, pain may be accompanied by sensory loss of the affected area [3], which is likely caused by progressive damage to the nerves induced by the growing tumor. However, tumors cells can release a variety of cytokines, chemokines and growth factors, which recruit immune cells. These tumor-associated immune cells acquire a tumor-supportive, antiinflammatory phenotype due to their interaction with tumor cells and release multiple additional signaling mediators [4,5]. This constant release of pro- and antiinflammatory mediators from tumors and tumor associated immune cells stimulates, amongst other things, angiogenesis ensuring survival and further growth of the tumor [6,7]. However, a variety of factors released by tumors can also sensitize or excite primary afferent neurons, causing the sensation of pain [6,8].

Moreover, peripheral nerves are composed of three distinct tissue compartments: the epineurium, perineurium, and endoneurium. The outermost epineurium surrounds a thin lamellated perineurium, which in turn surrounds axons, fibroblasts and Schwann cells, the most abundant cells of the endoneurium. Proteases such as cathepsin or metallopreinases, which can be released by macrophages and tumor cells, can damage these barriers that surround and protect peripheral nerves [9,10]. To identify pathomechanisms underlying tumor-induced pain states, a number of animal models have been established, which mimic cancer-induced pain [11]. One interesting model for cancer-induced pain that was previously outlined is based on the injection of MethA tumor cells in the proximity of the sciatic nerve [12]. This model allows the study of the progressive effects of tumors on a clearly defined area in the neighboring sciatic nerve. The nociceptive behavior recorded during tumor growth showed an increased thermal and mechanical hypersensitivity as well at later time points a mechanical hyposensitivity, which mirrors the sensory loss observed in patients. However, so far an investigation of the pathomechanisms underlying the observed pain responses has not been carried out.

Here, we aimed to investigate whether or not common pathomechanisms underlie the formation of tumor-induced pain. Therefore, we used three different cancer cell lines (E0771, MC57 and B16-F10) representing different cancer types (adenocarcinoma and fibrosarcoma, melanoma, respectively) [13,14,15] to induce tumor growth in the muscle tissue in proximity of the sciatic nerve. Importantly, the quality of the nociceptive responses induced by the different tumors was similar but independent of physical contact or tumor cell invasion. Instead, all tumors induced the accumulation of macrophages and dendritic cells at the outside of the sciatic nerve, which coincided with the appearance of allodynia. Later, microlesions formed in the outer layers of the sciatic nerve and loss of neural function occurred. Thus, despite the differences between the three cancer models, the results show a common pathology based on an inflammatory response underlying the development of the cancer-induced nociceptive behavior.

## 2. Materials and Methods

### 2.1. Animals

C57BL/6N mice were supplied by Janvier (Le Genest, France). The animals were cared for corresponding to International Association for the Study of Pain guidelines (Grant FK1065). For all experiments, the ethics guidelines for investigations in conscious animals were obeyed and the procedures were approved by the local ethics committee. All animals were tested at an age of 8–10 weeks. The animals had free access to food (Sniff standard diet) and water and were maintained in climate- (23 + 0.5 °C) and light-controlled rooms (light from 6.00 a.m. to 6.00 p.m.).

### 2.2. Cell Culture 

MC57 and B16-F10 cells were grown in DMEM (12 mM L-glutamine) supplemented with 10% FCS and 1% penicillin/streptomycin. E0771 cells were grown in RPMI 1640 (GIBCO, Carlsbad, CA, USA) with GlutaMAX-1 supplemented with 10% FCS, 1% penicillin/streptomycin, 1% sodium pyruvate, 1% non-essential amino acids. All cell lines were grown at 37 °C with 5% CO_2_ under humidified conditions.

### 2.3. Injection of Tumor Cells

The adenocarcinoma E0771 and fibrosarcoma MC57 were injected by inoculating (20–80 µL) 10^6^ viable tumor cells in cold PBS intramuscularly near the sciatic nerve (N. ischiadicus) into the left hind leg of C57BL/6N mice. The melanoma B16-F10 tumor cells were injected with 5 × 10^5^ viable tumor cells in cold PBS. For sham operated mice the hind leg was opened and the sciatic nerve was exposed with injection of cold PBS.

### 2.4. Behavioral Tests

The observer was unaware of the treatments in all tests. All mice were observed daily for signs of spontaneous pain starting at day 3. Therefore mice were placed in a plastic cage at room temperature. After an acclimation period of 30–60 min in the plastic cage, the mice were observed for 10 min and the duration of lifting or licking of the hind limbs was measured.

Mechanical thresholds were determined on the plantar side of a hind paw using a plantar aesthesiometer (Dynamic Plantar Aesthesiometer, Ugo Basile, Comerio, Italy). In this test a steel rod (2 mm in diameter) was pushed against the paw with increasing force (0 to 5 g over a 10-s period, time resolution 0.1 s) until a strong and immediate withdrawal occurred. Thermal thresholds on the plantar side of the hind paw were determined using the radiant heat test. Paw withdrawal latencies to radiant heat stimulation were measured in both paws. The intensity of the radiant heat was adjusted so that the paw withdrawal latency of a normal mouse falls in the range of 8–12 s. The paw withdrawal latency (against mechanical and thermal stimuli) was taken to be the mean of at least 5 consecutive trials with at least 20 s in between.

### 2.5. Electron Microscopy

The sciatic nerves of the ipsilateral and contralateral side were taken and fixed in 4% paraformaldehyde and 0.5% glutaraldehyde (EM grade) in 0.1 M sodium cacodylate buffer. Nerves were sectioned on a vibratome, embedded and serially sectioned based on an established EM-protocol with minor modifications [16].

### 2.6. Conventional Immunohistochemistry

On different time points after tumor cell implantation, the animals were killed by an overdose of carbon dioxide, and the sciatic nerves were extracted. The sciatic nerves were placed in tissue TEK cryo molds, dowsed with tissue TEK O.C.T freezing compound (both from Sakura Finetek, Torrance, CA, USA) and frozen in liquid nitrogen. Afterward, cryosections of 7 µm thickness were prepared using the cryotome Leica CM3050S (Leica, Wetzlar, Germany). The tissue was fixed for 10 min in −20 °C methanol followed by 1 min in acetone, then permeabilized with 0.025% Triton in PBS 2 × 5 min and blocked for 90 min in 3% BSA and 10% goat serum in PBS. For staining, the tissue was incubated with ZO-1 antibody (617300, Sigma-Aldrich, Deisenhof, Germany) or Claudin-1 (ab15098, Abcam Cambridge, UK) followed by a Cy3-labeled anti-rabbit antibody (Sigma-Aldrich, Deisenhof, Germany). The Image J Software version 1.50i (National Institutes of Health, Bethesda, MD, USA) was used to determine the signal intensity in a 250 pixel range on the surface of sciatic nerves. Values under 50% of the maximal intensity were defined as decreased/absent signal. 4 images of each sciatic nerve were analyzed and the mean was used for calculation using Graph Pad Prism 5 (Graph Pad Software, San Diego, CA, USA).

### 2.7. Multi Epitope Ligand Cartography (MELC)

The MELC technology is an immunohistological imaging method that allows the visualization of 20–40 proteins on the same sample and has been described previously [17,18]. Briefly, tissues from the various inflammation models were embedded in tissue freezing medium (Leica Microsystems, Nussloch, Germany), cryosections of 7 µm thickness were applied on silane-coated coverslips, fixed in 4% paraformaldehyde in PBS for 15 min, permeabilized with 0.1% Triton X100 in PBS for 15 min and blocked with 3% BSA in PBS for 1 h. The sample was placed on the stage of a Leica DM IRE2 and a picture was taken. Then, by a robotic process, the sample was incubated for 15 min with bleachable fluorescence-labelled antibodies (Supplementary Table S1) and rinsed with PBS. Afterward, the phase contrast and fluorescence signals were imaged by a cooled charge-coupled device camera (Apogee KX4; Apogee Instruments, Roseville, CA, USA, 2048 × 2048 pixels; final pixel size was 286 nm × 286 nm). After the MELC staining was completed the tissue sample was stained using Diff-Quick according to the manufacturer’s instructions (Dade Behring, Marburg, Germany).

To delete fluorescence signals, a bleaching step was performed and the post-bleaching image was recorded. Then the next antibody was applied. For data analysis using the corresponding phase contrast images, fluorescence images produced by each antibody were aligned pixel-wise and were corrected for illumination faults using flat-field correction. The post-bleaching images were subtracted from their following fluorescence image.

### 2.8. Fluorescence Activated Cell Sorting (FACS)

Polychromatic flow cytometry was performed essentially as described previously [17]. Briefly, single-cell suspensions were generated from solid tissues by digestion with 3 mg/mL Collagenase IA (Sigma, Steinheim, Germany) DMEM for 45 min at 37 °C, followed by filtration through a 70 µm nylon mesh (BD Biosciences, Franklin Lakes, NJ, USA). Then the samples were incubated for 5 min in DMEM containing 10% FCS to stop the lysis followed by incubation in ACK buffer for 5 min. The incubation with an antibody cocktail was performed for 1 h at room temperature. Cells were transferred to fluorescence activated cell sorting (FACS) tubes. Samples were acquired with a LSRII/Fortessa flow cytometer (BD Biosciences, Heidelberg, Germany) and analyzed using FlowJo software 7.6.5 (Treestar, Ashland, OR, USA). For gating, fluorescence minus 1 (FMO) controls were used. The instrument calibration was controlled daily using Cytometer Setup and Tracking beads (BD Biosciences).

### 2.9. Microparticle Analysis

Fluorescent labeled microparticles (0.06 and 3.6 µm diameter) (Kisker Biotech, Steinfurt, Germany) were used to investigate the permeability of the outer layers of the ipsilateral and contralateral sciatic nerves (perineurium). After sacrificing the animals, the skin was opened and the muscle tissue above the sciatic nerve removed. The nanoparticles were suspended in PBS (1:200) and 100 µL were applied on the nerve every 2.5 min for a total of 10 min at room temperature. Then the tissue was prepared as described above for FACS analysis. The number of microparticles in the nerves was determined by FACS and compared with untreated control nerves.

### 2.10. LC-MS Analysis

The semitargeted LC-QTOFMS analysis was performed as previously published [19]. Briefly, the tissue samples were processed using wet grinding on a Mixer Mill MM 400 (Retsch, Haan, Germany) with a frequency of 25/s for 2.5 min. The tissue concentrations were adjusted to 0.007 mg/µL with water: ethanol (3:1, *v/v*) and four zirconium dioxide grinding seeds were added. To 145 µL of the resulting homogenate 150 µL internal standard solution in methanol and 500 µL MTB E (Carl Roth, Karlsruhe, Germany) were added. All internal standards (Supplemental Table S2) were purchased from Avanti Polar Lipids (Alabaster, AL, USA). After centrifugation at 20,000× *g* for 5 min, the lower phase was reextracted using 200 µL of MTBE: methanol: water (10:3:2.5, *v/v/v*). The upper organic phases were combined, split into two aliquots for measurement in both ionization modes and dried at 45 °C under a nitrogen stream. Before the analysis, samples were reconstituted with 120 µL methanol. Additionally, pooled homogenates were used as quality control samples with two injections at the beginning, the middle and the end of the run. The samples were analyzed on a TripleTOF 6600 (Sciex, Darmstadt, Germany) coupled to a Nexera-X2 (Shimadzu Corporation, Kyoto, Japan) using positive and negative electrospray ionization. The analyte separation was achieved on a Zorbax RRHD Eclipse Plus C8 1.8 µm 50 × 2.1 mm ID column (Agilent, Waldbronn, Germany) using a binary gradient. For compound detection, a mass range from 100 to 1000 *m*/*z* was scanned and six data-dependent spectra were acquired per cycle. The data were acquired using Analyst TF v1.71 and peaks were integrated with MultiQuant v3.02 (both from Sciex), using one internal standard per lipid class for normalization. Compounds were identified as described previously using MasterView v1.1 (Sciex) with a 5 ppm mass tolerance, isotopic distribution and the information obtained from the MS/MS spectra [19].

### 2.11. Multiplex Cytokine Assay

Cytokine and chemokine levels were determined in tumors and the sciatic nerve using the Mouse Cytokine/Chemokine bead immunoassay kit, (ProcartaPlex Human kits, eBioscience, San Diego, CA, USA). Tissue samples were frozen directly at −80 °C until they were used for LUMINEX measurement. Nerves and tumors were lysed in 400 µL lysis buffer (50% PhosphoSafe and 50% Protease inhibitor cocktail (Merck, Darmstadt, Germany). Samples were cut in small pieces and then sonicated once at 60% for 10 s. Afterwards all samples were centrifuged for 10 min at 10.000× *g*. In 50 µL of the samples the concentrations of VEGF-A, IFN-gamma, IL-1alpha, IL-1beta, IL-10, IL-4, IL-5, IL-6, IL-9, Leptin, M-CSF, MIP-1alpha, MIP-1beta, RANTES, TNF-alpha and MCP-1/CCL2 were determined using a Bioplex 200 (BioRad, Hercules, CA, USA).

### 2.12. Statistical Analysis

Statistical significance was determined by a Student’s *t*-test, one-way ANOVA using a Bonferroni post-hoc-test, or Kruskal-Wallis one-way ANOVA with Dunn’s post-test through the GraphPad Prism 5 software as outlines in the figure legends.

## 3. Results

### 3.1. All Tumor Types Induce Hypersensitivity and Late-Stage Hyposensitivity

The cancer cell lines E0771 (adenocarcinoma), MC57 (fibrosarcoma) and B16-F10 (melanoma) were injected in the muscle tissue in direct proximity of the sciatic nerve. These three cell lines were originally generated in C57BL/6 mice [13,14,15] and therefore were also studied by us in this mouse strain. Following the tumor cell injection the mice in the different tumor groups did not show signs of spontaneous pain. The health status of the mice was monitored on a daily basis whereby none of the mice showed changes in the posture, movements, response to handling, general behavior or fur appearance (Supplementary Table S3). Most importantly, the body weight of the mice did not decrease during the duration of the experiment (Supplementary Figure S1).

**Figure 1:**
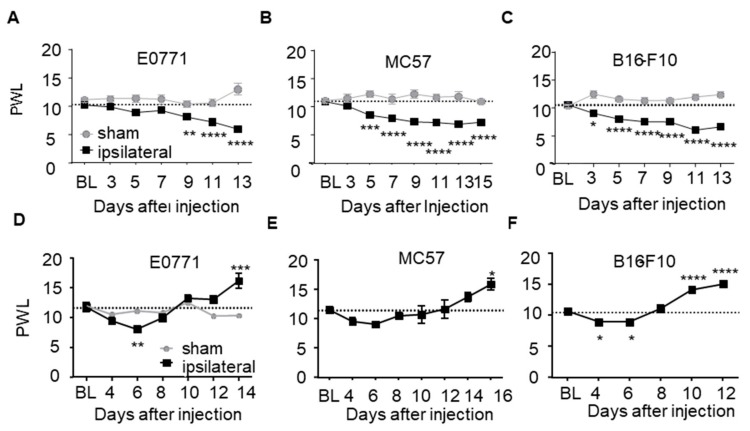
Tumor growth next to the sciatic nerve induces mechanical allodynia and thermal hyposensitivity. (**A**–**C**) Mechanical paw withdrawal latencies in mice bearing E0771 (**A**; *n* = 12), MC57 (**B**; *n* = 9) and B16-F10 (**C**; *n* = 10) tumors. (**D–F**) Thermal paw withdrawal latencies in mice bearing E0771 (**D**; *n* =8–11), MC57 (**E**; *n* = 9) and B16-F10 (**F**; *n* = 5–10) tumors. Data are shown as mean ± S.E.M., One-way ANOVA/Dunnett’s test vs. baseline. * *p <* 0.05, ** *p <* 0.01, *** *p <* 0.001, **** *p <* 0.0001.

However, the mice developed a progressive allodynia starting at day 3, 5 and 9 after injection of B16-F10, MC57 and E0771 tumor cells, respectively (Figure 1A–C). Interestingly, paw withdrawal latencies in response to thermal stimulation significantly decreased only in E0771 tumor bearing mice (day 6 after cell injection) (Figure 1D–F). Notably, at later time points the mice developed a thermal hyposensitivity in all tumor groups which were significantly elevated as compared to baseline after 10 (B16-F10), 14 (E0771) and 16 days (MC57) (Figure 1D–F). The observed late phase hypoalgesia is indicative for the loss of function of thermosensitive neurons, which mirrors the sensory loss observed in patients.

**Figure 2:**
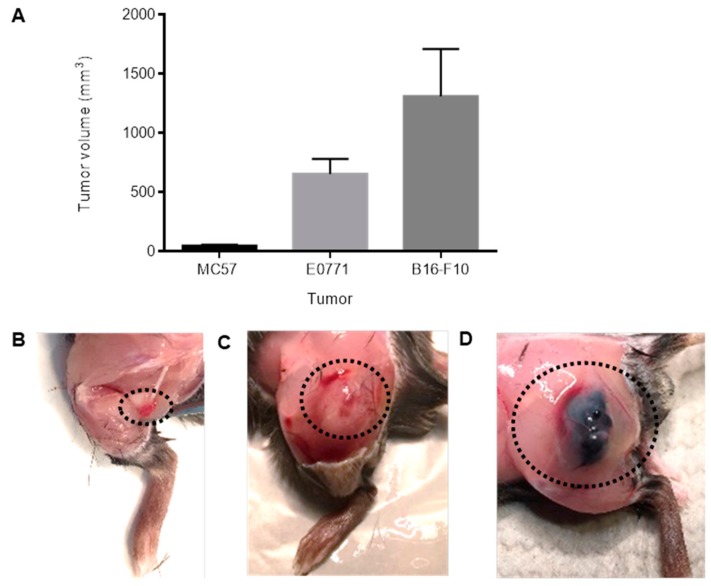
The tumor volumes differ strongly between the three tumor types. (**A**) Tumors were taken and their volumes were determined when a significant hypoalgesia was observed. MC57: day 19, *n* = 5, E0771: day 14, *n* = 14, B16-F10: day 13, *n* = 5, Data are shown as mean ± S.E.M. (**B**–**D**) Representative images of MC57 (**B**), E0771 (**C**) and B16-F10 (**D**) tumors. The dotted areas outline the position of the tumors.

Next, at the time point when a significant hypoalgesia was observed (MC57: 19 days, E0771: 14 days and B16-F10: 13 days after tumor cell injection) tumor volumes were determined. Notably, MC57-tumors (49 ± 8.8 mm^3^) were 13 times smaller than E0771-tumors (654 ± 126 mm^3^) and 27 times smaller than B16-F10-tumors (1311 ± 398 mm^3^), respectively (Figure 2A–D). Thus, since mice bearing the small-sized MC57 tumors showed an earlier onset of the decrease in the mechanical paw withdrawal latencies as mice bearing the much bigger E0771 tumors, the data show no correlation between hyper- and hyposensitivity and tumor size. In addition, MC57 tumors were during the first 14 days too small to come in direct contact with the sciatic nerves, therefore compression or bending of the sciatic nerve can be ruled out as reason for the development of sensory hypersensitivity.

### 3.2. Tumor Cells Do Not Infiltrate the Sciatic Nerves

**Figure 3:**
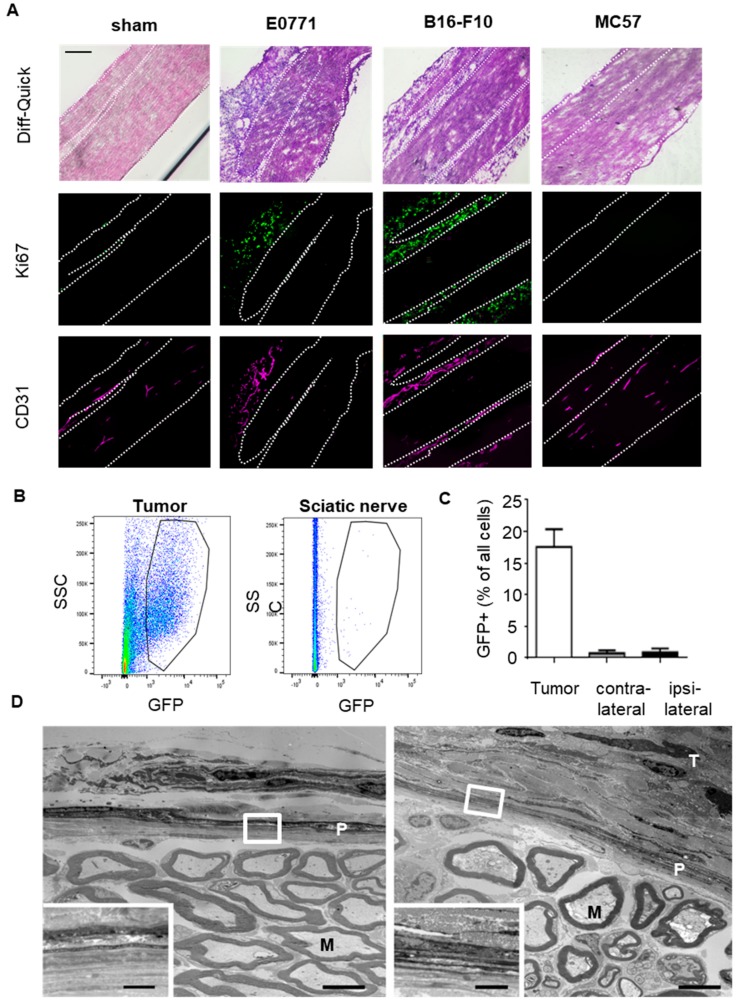
Tumor cell invasion of sciatic nerves is not seen for any of the three tumor types. (**A**) Immunohistological MELC staining of sciatic nerves from sham operated mice and mice with E0771 (d14), B16-F10 (d13) or MC57 (d19) tumors. Shown in false colors are marker for proliferating cells (Ki67) and endothelial cells (CD31). White dotted lines depict borders of the sciatic nerve. All areas are distal of splitting to nervus tibialis, peroneus and suralis. The black bar represents 100 µm. (**B**,**C**) FACS analysis of tumors and sciatic nerves from mice injected with GFP-overexpressing E0771 cells (d14). Number of GFP-expressing cells in tumor, ipsilateral and contralateral nerves shown as mean ± S.E.M. (*n* = 5). (**D**) Electron microscopic image of ultrathin cross-sections of sciatic nerves from sham-operated mice (left panel) and 14 days after implantation of E0771 cells (right panel). Scale bars 5 µm. The white squares depict the area magnified in the lower left corners (scale bars 1 µm). M, example of a myelinated axon; P, perineurium; T, tumor area.

To determine whether or not tumor cell invasion of the sciatic nerves might be the reason for the nociceptive response to the tumors, we stained the sciatic nerves for the presence of tumor cells. Therefore we harvested the nerves with the attached tumors (MC57 19 days, E0771 14 days and B16-F10 13 days after tumor cell injection) and stained the tumors using the proliferation marker Ki67. It should be noted that it was not possible to harvest MC57 tumors attached to the sciatic nerves, since they were due to their small size not in direct contact with the sciatic nerve. The attached E0771 and B16-F10 tumors showed a strong vascularisation (CD31) and proliferation (Ki67). However, no signal was detected in sciatic nerves from naïve or tumor bearing mice (Figure 3A). The tumors were identified besides the Ki67 staining also by a strong vascularization, as seen by CD31-staining of endothelial cells. In addition we employed GFP-overexpressing E0771 cells to quantify the amount of tumor cells in the nerves using FACS analysis. We found a strong GFP signal in cells isolated from tumors but not in sciatic nerves, which were excised in the proximity of the tumors (Figure 3B,C). Also, electron microscope images showed no gross morphological changes of the sciatic nerve area adjacent to the tumor tissue (Figure 3D). Hence, tumor cell infiltration of the sciatic nerve does not seem to occur in the tumor bearing mice.

### 3.3. Tumors Induce Microlesions in the Neighboring Sciatic Nerves

**Figure 4:**
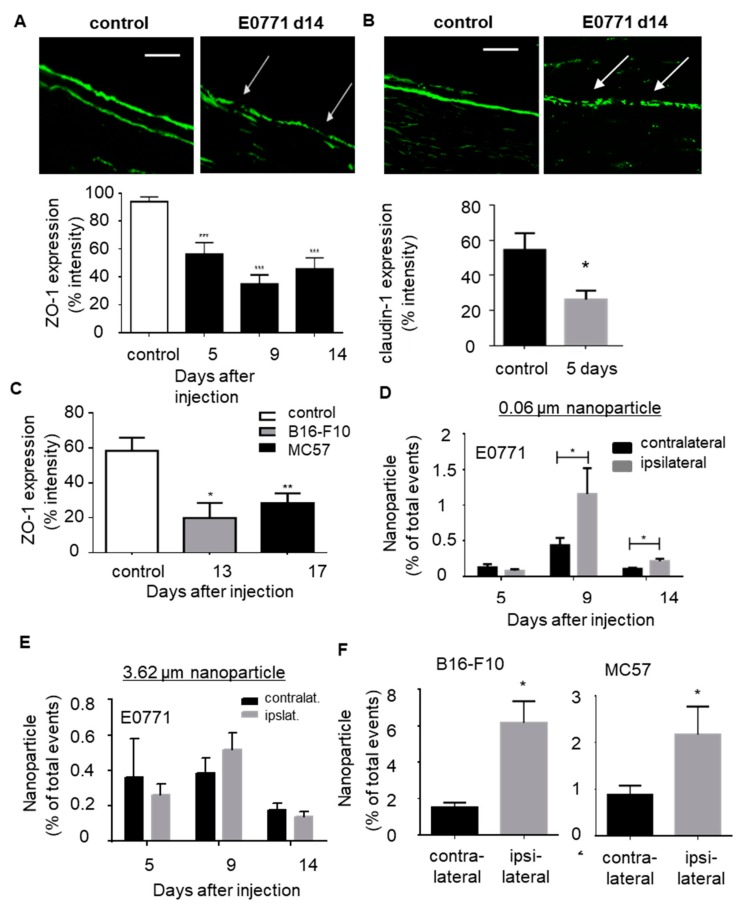
Tumor growth induces microlesions in the sciatic nerves. (**A**,**B**) Immunohistological staining for tight junction marker ZO-1 (panel A) and claudin-1 (panel B) in sciatic nerves from naïve mice and 14 days after E0771 cell injection. White arrows depict ZO1 downregulation in an ipsilateral nerve. Scale bar 100 µm. Quantification of ZO-1 and claudin-1 staining in sciatic nerves at different time points (panel A) or claudin-1-staining 5 days after E0771 injection (panel B). Data are shown as mean ± S.E.M. (control *n* = 12, 5 days *n* = 4, 9 days *n* = 4 and 14 days *n* = 4). One way ANOVA, *** *p* < 0.001. (**C**) Quantification of ZO-1 staining in sciatic nerves after B16-F10 (14 days) and MC57 (18 days) injection. Data are shown as mean ± S.E.M. Control *n* = 12, B16-F10 *n* = 4 and MC57 *n* = 8. One way ANOVA, * *p* < 0.05, ** *p <* 0.01. (**D,E**) FACS analysis of fluorescent labeled particles with an average size of 0.06 µm (panel E) or 3.6 µm (panel F) in contralateral and ipsilateral sciatic nerves at different time points after E0771 tumor cell injection. Data are shown as mean ± S.E.M. (*n* = 6). Two-tailed Students *T*-test * *p* < 0.05. (**F**) Same as panel E except that the amount of particles in sciatic nerves 14 days after B16-F10 or 18 days after MC57 cell injection. Data are shown as mean ± S.E.M. (*n* = 6) * *p* < 0.05.

In the next step we tested whether or not the perineural layers of the sciatic nerves are affected by the neighboring tumors. Since the electron microscope images did not show major damage or tumor cell infiltration through the perineurium (Figure 3D), we focused on the integrity of tight junctions, which are essential for the barrier function of the perineurium. Immunohistochemical staining of the tight junction protein ZO-1 showed an increased occurrence of regions with downregulated ZO-1 expression in the perineurium. This altered distribution was seen starting 5 days after E0771 tumor cell injection (Figure 4A), at the first time point with significantly decreased mechanical and thermal paw withdrawal latencies. At this time point also the tight junction protein claudin-1 was significantly downregulated (Figure 4B). Likewise, mice bearing B16-F10 and MC57 tumors showed downregulation of ZO-1 expression in the perineurium (Figure 4C).

Next, we investigated whether or not the observed altered expression of the perineurial tight junctions has physiological consequences on the barrier function. Therefore we incubated fluorescence-labeled particles with an average size of either 0.06 µm or 3.6 µm with the contra- and ispsilateral sciatic nerves 5, 9 and 14 days after E0771 tumor cell injection.

The particles were applied on the exposed sciatic nerves for 10 min before the nerve was excised and prepared for FACS analysis. A significant increase of particles with an average size of 0.06 µm (Figure 4D), but not with a size of 3.6 µm (Figure 4E), was seen in sciatic nerves 9 and 14 days after E0771 tumor cell injection. A similar increase in the uptake of 0.06 µm sized particles was observed with sciatic nerves taken after injection of B16-F10 (14 days) and MC57 (18 days) tumor cells (Figure 4F). Thus, all three tumor types induce downregulation of tight junctions in the perineurium followed at a later time point by the appearance of microlesions with a size between 0.06 and 3.6 µm.

**Figure 5:**
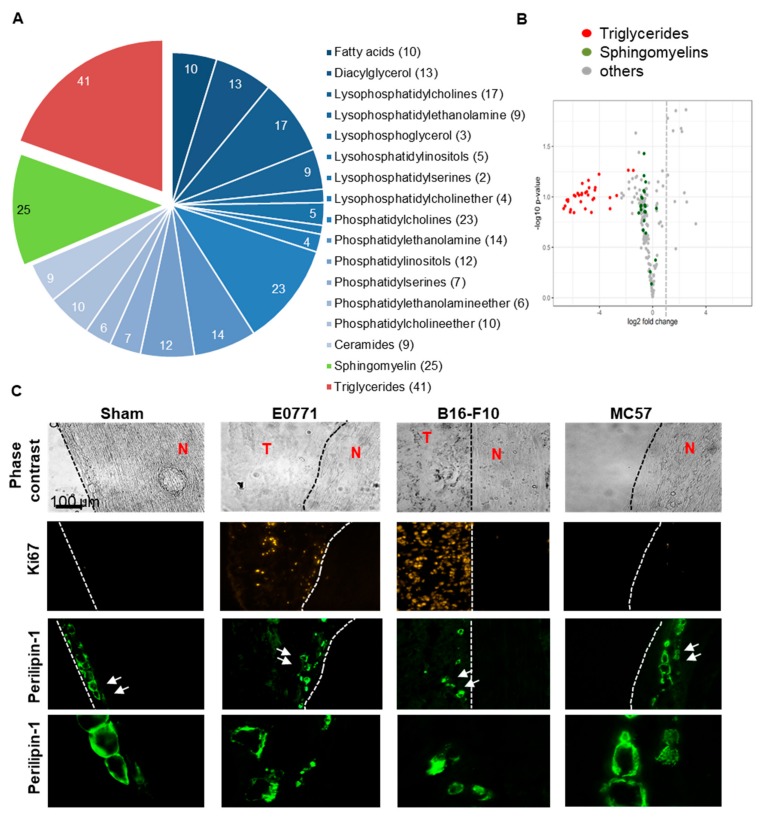
Localization and lipid content of epineural adipocytes is changed by neighboring tumors. (**A**) Lipidomic comparison of ipsi- and contralateral sciatic nerves 14 days after E0771 tumor cell injection (*n* = 5). Shown are lipid classes of the 206 analyzed lipids. Numbers in the chart indicate the number of different lipids detected per lipid class. (**B**) Vulcano plot showing the regulation of the lipids in the sciatic nerves from the ipsilateral side in comparison to the contralateral side. (**C**) Immunohistochemical images showing adipocytes located in the outer layers of sciatic nerves and Ki67 staining for proliferative cells. Adipocytes in sciatic nerves injected with E0771 (d14), B16-F10 (d9) or MC57 (d19) show irregular perilipin-1 staining. The white arrows show cells, which are magnified in the bottom panel. White arrows depict adipocytes, where a shrinking of the cell or an even distribution of perilipin-1 throughout the cell is visible. T, tumor tissue; N, sciatic nerve.

Since epineural adipocytes are discussed to have an important protective role in peripheral nerves, we performed a semi-quantitative untargeted lipidomic analysis of the sciatic nerves. Here, we compared the lipidome of sciatic nerves from the ipsi- and contralateral sides of mice 14 days after E0771 tumor cell injection. The 206 analyzed lipids in the screen represented 17 lipid classes from which the triglycerides and the sphingomyelins had the most members (Figure 5A). Comparison of the lipid amounts in ipsi- and contralateral nerves showed on the ipsilateral side a strong downregulation of long-chain sphingomyelins (chain length of ≥36) in average by 40% ± 0.01 (*p* ≤ 0.0001, two-tailed Students *T*-test) and of the 41 detected triglycerides by in average 95% ± 0.01 (*p* ≤ 0.0001, two-tailed Students *T*-test; Figure 5B; Supplementary Figures S2 and S3A). While the decreased amounts of the sphingomyelins points towards damage in neuronal myelin sheaths, the dramatic loss of triglycerides suggests a loss of epineural adipocytes and decreased energy metabolism. Fatty acids are transported into mitochondria via their corresponding carnitine ester. There acylcarnitines are converted back into free carnitine and long-chain acyl-CoAs, which can then be oxidized [20]. Fittingly, also the detectable acylcarnitines (14:1, 16:0, 18:0, 18:1 and 18:2) were decreased in the ipsilateral nerve in average by 54 ± 3.7% as compared to the contralateral side (*p* ≤ 0.001, two-tailed Students *T*-test; Supplementary Figure S3B).

Since the lipidomic data suggested a triglyceride deficiency in adipocytes, we visualized these cells in the sciatic nerves using perilipin-1 staining. In adipocytes, perilipin forms a barrier at the surfaces of lipid droplets that restricts the access of cytosolic lipases to the lipids, thus promoting triacylglycerol storage [21,22]. In sciatic nerves from sham operated mice adipocytes were located in the outer layers of the nerves and showed an even perilipin 1 distribution around the lipid droplets (Figure 5C). In nerves isolated from mice 14 days after E0771 tumor cell injection adipocytes are partly shrunken or perilipin-1 was distributed throughout the cells (Figure 5C). Similar observations were made in nerves from mice injected with MC57 or B16-F10 tumor cells. Importantly, in areas where the tumor (represented by Ki67 staining) is in contact with the sciatic nerve (E0771 and B16-F10) the adipocytes seem no longer to be attached to the epineurium (Figure 5C). Notably, the adipocytes were in vicinity of proinflammatory macrophages (CD86^+^/F4 80^+^) and dendritic cells (CD86^+^/F4 80^+^) (Supplementary Figure S4). This proinflammatory environment might lead to damage of the epineurium and subsequently detachment of the adipocytes. Thus, taken together the data show that the outer layers of the sciatic nerves are damaged by the neighboring tumors without that tumor cell invasion or direct contact (i.e., MC57 tumors) seem to play a role.

### 3.4. Tumor-Dependent Inflammation of the Sciatic Nerves

To investigate whether or not inflammatory cells are found outside of sciatic nerves in mice injected with E0771 tumor cells, we performed a MELC analysis, which is an automated technology for multiple sequential immunohistochemistry, allowing the sequential imaging with 30–40 antibodies on the same tissue sample. Here we used antibodies to identify specific immune cell types including T-cells, (CD4, CD8), dendritic cells (MHC II, CD11c) macrophages (F4 80), eosinophils (Siglec F) and neutrophils (Ly6G) as well as more general immune cell markers (CD45, CD11b).

**Figure 6:**
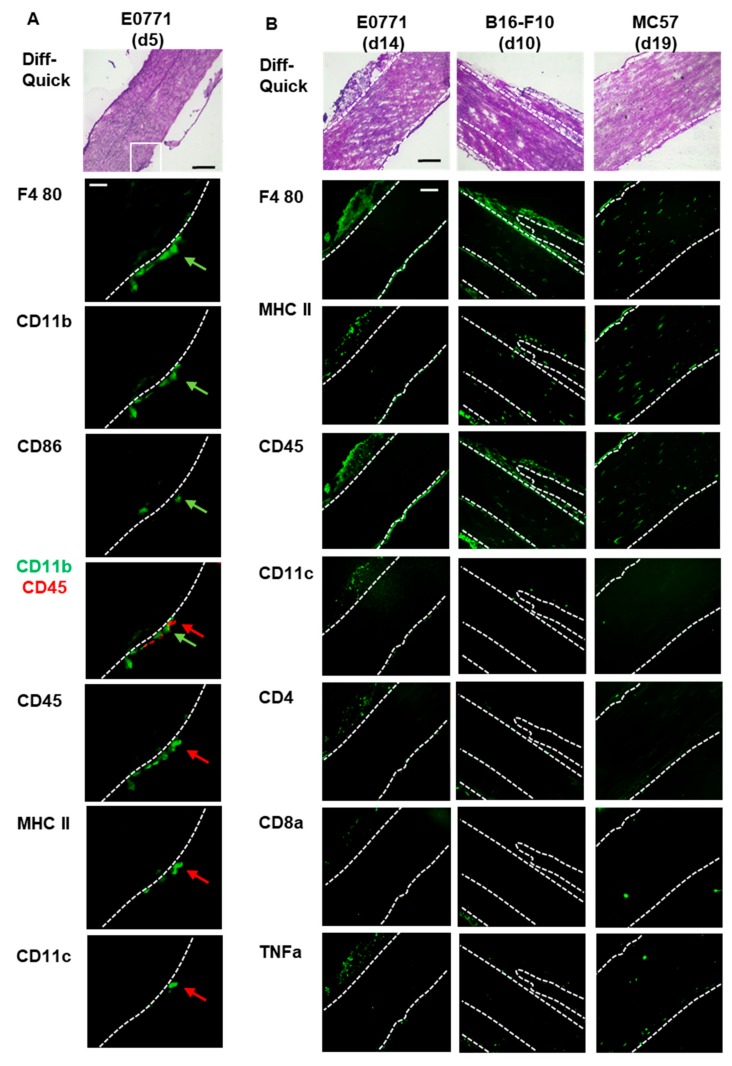
Tumor-induced immune cell recruitment to sciatic nerves. (**A**) MELC staining of sciatic nerves from mice 5 days after E0771 tumor cell injection shows macrophages and dendritic cells at the outer layers of the sciatic nerve. The white square depicts the area shown in the MELC stainings below. Table 100. µm and the white bar 25 µm. The green arrow depicts a proinflammatory macrophage (F4 80+/CD11b+/CD86+) and the red arrow a dendritic cell (MHC II+/CD45+/CD11c^−^). (**B**) Immune cell localization after E0771 (d14), B16-F10 (d10) or MC57 (d19) tumor cell injection. The black bar represents 100 µm. White dotted lines depict borders of the sciatic nerve.

Additionally, antibodies against CD86 and TNFα were included to identify proinflammatory cells (Supplementary Table S1). In sciatic nerves from naive mice no immune cells were detected inside or at the outside of the sciatic nerve (Supplementary Data S5). 5 days after E0771 cell injection, the time point when mechanical hypersensitivity is first detected, macrophages (F4 80^+^/CD11b^+^/CD45^−^), which were partially proinflammatory (CD86^+^), as well as dendritic cells (CD11c^+^/CD45^+^/MHC II^−^ and CD11c^−^/CD45^+^/MHC II^+^) appeared at the outside of the sciatic nerve (Figure 6A). Other immune cells such as T-cells, neutrophils, eosinophils, T-cells (CD4 and CD8) or B-cells (data not shown) were not detectable. In sciatic nerves from naïve mice no macrophages or dendritic cells were observed (Supplementary Figure S5).

14 days after E0771 injection in addition to the macrophages and dendritic cells low numbers of T-cells (CD4 and CD8) were seen at the outside of the sciatic nerve (Figure 6B). Again neutrophils, eosinophils and B-cells were not detected. Similar observations were obtained 10 days after B16-F10 and 19 days after MC57 injection (Figure 6B). Interestingly, at this late time pointes macrophages (F4 80^+^) and dendritic cells (CD45^+^/MHC II^+^/CD11c^−^) appeared also inside the sciatic nerve (Figure 6B). Positive TNFα staining points suggests a proinflammatory immune reaction in and around the sciatic nerve. Staining for F4 80 and CD86 (M1 macrophages) as well as F4 80 and CD206 (M2 macrophages) showed for mice injected with E0771 tumor cells that, as expected, CD206-expressing M2 macrophages dominate the tumor tissue, while in areas close to the sciatic nerve also CD86^−^ expressing M1 macrophages are seen (Supplementary Figure S6). Similar observations were made in mice injected with B16-F10 tumor cells although here at the late stage M1 macrophages were also seen within the sciatic nerve. Likewise, in mice injected with MC57 tumor cells M1 macrophages were located within the sciatic nerve (Supplementary Figure S6) suggesting an ongoing inflammation in the sciatic nerve.

**Figure 7:**
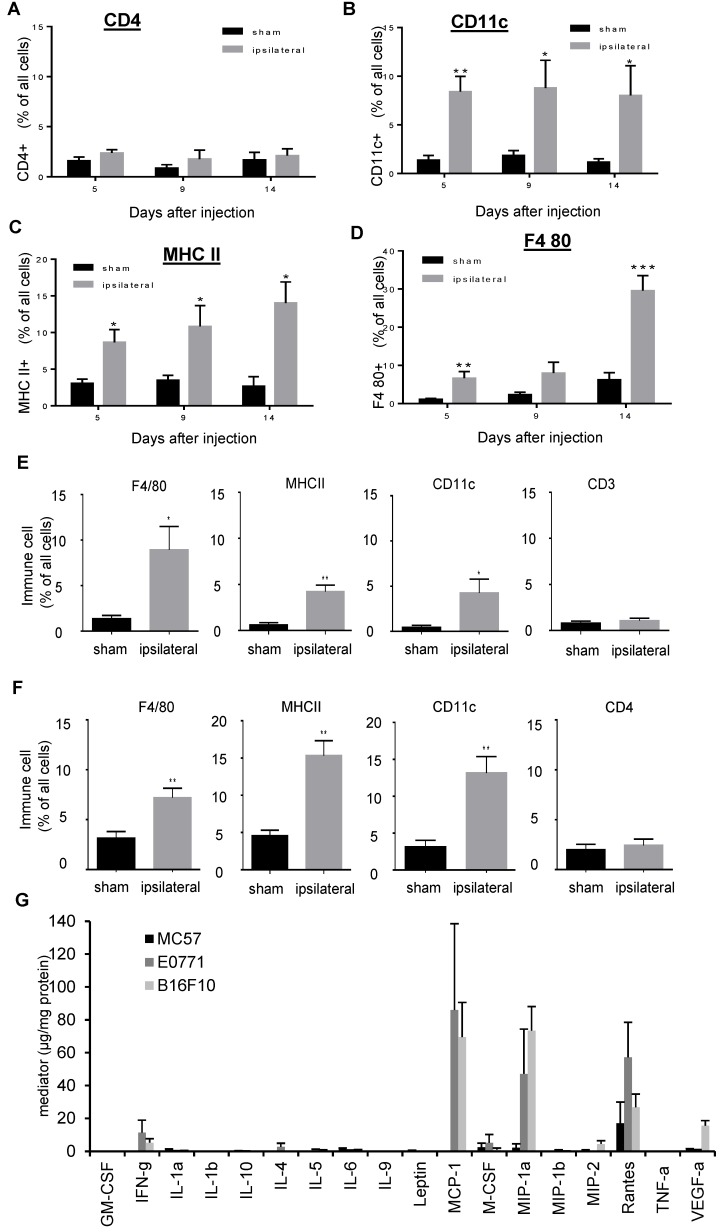
Macrophages and dendritic cells are recruited to the sciatic nerves. (**A**–**D**) FACS analysis of CD4 (Panel A), CD11c (panel B), MHC II (panel C), F4 80 (panel D) positive cells in contralateral and ipsilateral nerves 5, 9 and 14 days after E0771 cell injection. Data are presented as mean ± S.E.M. (contralateral *n* = 4–7, ipsilateral *n* = 5–14), Two-tailed Student *t* test, * *p* < 0.05, ** *p* < 0.01, *** *p* < 0.001. (**E,F**) Same as Panel A and B except that cells were determined 19 days after MC57 (panel E) or 10 days after B16-F10 (panel F). (**G**) Cytokine and chemokine levels were detected by multiplex cytokine/chemokine assay in E0771 (d6), MC57 (d18) and B16-F10 (d9) tumors.

The immunohistological MELC data were validated by FACS analysis of sciatic nerves isolated from mice at different time points after E0771 tumor cell injection. Here, increasing amounts of macrophages and dendritic cells but not of T-cells were observed (Figure 7A–D). A similar immune cell recruitment was observed for sciatic nerves isolated from mice 19 days after MC57 (Figure 7E) or 10 days after B16-F10 (Figure 7F) tumor cell injection. Since the data suggest that the tumor growth is causing the recruitment of proinflammatory dendritic cells to the outside of the sciatic nerves, we determined the production of various cytokines and chemokines in these tumors. Most interestingly, E0771 and B16-F10 tumors showed high levels of the chemokines MCP-1 (CCL2), MIP-1α (CCL3) and RANTES (CCL5) (Figure 7G). In the much smaller MC57 tumors only RANTES was detectable (Figure 7G). Thus all three tumor types are producing at least one chemokine, which is able to induce chemotactic migration of macrophages and dendritic cells [23,24]. Thus the three tumor types are expressing chemokines capable to recruit macrophages and dendritic cells. The appearance of these cells on the outside of the sciatic nerve correlates with the appearance of hypersensitivity and the disturbance of perineural tight junctions.

## 4. Discussion

The treatment of tumor-induced neuropathic pain in patients with primary tumors or metastases growing in the proximity of peripheral nerves poses a severe therapeutical problem. The reasons for this tumor-induced neuropathic pain are mostly unknown, but are often attributed to a mechanical compression or tumor cell invasion of the affected peripheral nerves. Here, we describe the pathophysiology and nociceptive responses in response to the growth of different tumor types (fibrosarcoma, melanoma and adenocarcinoma) [13,14,15] in proximity to the sciatic nerve. We found for all three tumor types common pathophysiological features including a similar development of nociceptive responses, an early appearance of macrophages and dendritic cells at the outside of the sciatic nerve and a concomitant disturbance of tight junctions. Subsequently microlesions appeared in outer layers of the sciatic nerve and additional immune cells appeared outside and partially inside of the damaged sciatic nerve.

Most interestingly, the nociceptive responses to mechanical and thermal stimuli were comparable for all three tumor cell types. The finding that the mice developed in all tumor groups a thermal hyposensitivity was surprising, since a previous report showed that in the same model MethA sarcoma cell tumors induced in BALB/C mice mechanical but not thermal hyposensitivity [11]. This discrepancy with our data indicates variable responses in regard to the loss-of-function of neuronal subtypes depending on the tumor type and/or the mouse strain. While these differences remain to be investigated, the nociceptive and cellular responses to tumor growth within one mouse strain were surprisingly consistent despite the different tumor origins and the different growth rates. In this regard it becomes obvious that mechanical compression of the nerves by the tumors can be ruled out as reason for the increased hypersensitivities. Especially the findings that MC57 tumors are even at a time point when thermal hyposensitivity is observed barely coming in physical contact with the sciatic nerves, makes nerve compression as primary reason for the increased nociception highly unlikely. Along this line MC57 tumors develop mechanical hypersensitivity earlier than the 13-times bigger E0771 tumors and as fast as the 27-times bigger B16-F10 tumors, which underlines that the tumor size does not correlate with the pain development.

However, we found that the first appearance of mechanical hypersensitivity coincided with the arrival of immune cells (macrophages and dendritic cells) and a disturbance of tight junctions in the perineurium. Shortly afterwards in the outer layers of the sciatic nerves microlesions with a size between 0.06 and 3.6 µm were detected. Smaller lesions might occur even earlier but could not be detected due to the lack of the availability of smaller fluorescent-labelled particles. These microlesions allow the penetration of the perineurium by small molecules released by tumors (e.g., adenosine, sphingosine-1-phosphate) as well as pronociceptive and proinflammatory mediators released by macrophages and dendritic cells (e.g., prostaglandins, cytokines) [8,25] at the outside of the sciatic nerves. Whether the observed demyelination is a primary effect or whether it is a secondary effect of axonal damage due to overstimulation of the neurons remains unclear.

The formation of the microlesions itself is most likely the result of multiple events. On one hand, the release of proteases by immune cells on the outside of the nerve can induce a primary damage of epi- and perineurium. In this regard it was shown in a model where tumor cells were injected inside the sciatic nerve, that preferentially macrophages promoted further nerve invasion through cathepsin B [9]. It should be noted that also other proteases, which are released by macrophages, including MMP2 and MMP9 [10], might play a role in tumor-induced damages of the sciatic nerve.

On the other hand we observed dramatically decreased triglyceride and acylcarnitine levels in the sciatic nerve. This points towards the depletion of the triglycerides from adipocytes within the peripheral nerves and may indicate even a loss of adipocytes in the sciatic nerve. There is mounting evidence that adipocytes are crucial for the integrity of peripheral nerves. For example, lipin1 is an enzyme expressed in adipocytes of the epineural compartment of adult nerves where it catalyzes the second last step in triglyceride synthesis. A loss-of-function mutation of lipin1 is sufficient to cause a demyelinating neuropathy in the peripheral nerve system [26]. Also, in a model for trauma-induced neuropathic pain, leptin, an adipokine produced by adipocytes, was shown to be critical for the development of the tactile allodynia [27]. Here, leptin released by epineural apdipocytes induced in recruited macrophages the production of pronociceptive mediators causing neuropathic pain. Moreover, it is discussed that the adipocytes are necessary for stabilizing energy metabolism of Schwann cells. In this regard it has been shown that suppression of the synthesis of fatty acids in Schwann cells causes epineural adipocytes to undergo lipolysis, suggesting a compensatory role for the epineural adipocytes [28]. However, it should be noted that under non-pathological conditions the ability of Schwann cells to provide myelin sheaths and preserve functional peripheral neurons, were not affected by the presence of epineural adipocytes with defective lipid metabolism [29]. Also, lipid metabolism in some tumors is regulated also by the availability of lipids in the microenvironment and that lipid metabolism and, more specifically, fatty acid metabolism contribute to tumorigenesis. In this regard it was shown for ovarian cancer that adipocytes provide fatty acids for the tumor cells enhancing tumor growth [30]. Thus, the decreased lipid content in the epineural adipocytes observed in our model might also be based on the increased metabolism and transport to tumor cells. 

Finally, we found that epineural adipocytes at the tumor/sciatic nerve interface were accompanied by proinflammatory macrophages and dendritic cells. The proinflammatory environment of the adipocytes might cause damage of the epineurium and lead to the observed detachment from the sciatic nerve. This detachment in combination with the proinflammatory environment could induce apoptosis or necrosis of the adipocytes, which would be reflected by the decreased triglyceride levels of the sciatic nerve as well as the changed morphology and decreased size of the adipocytes.

Thus the recruitment of inflammatory cells to the outside of the sciatic nerve might start a chain of events that leads to damage of the epineurium and depletion of triglycerides from epineural adipocytes. Since at the same time pronociceptive and proinflammatory mediators can enter the sciatic nerve through the microlesions, Schwann cells might not be able any longer to support the formation of myelin sheaths of myelinated neurons. Similarly non-myelinating Schwann cells might be unable to protect the non-myelinated neurons, causing in the end neuronal death leading to the observed loss-of-function of thermal sensitivity. Therapeutically, interventions aiming to suppress inflammation of the sciatic nerve or to stabilize the perineurium and the epineurium barrier function might offer a possibility to prevent tumor-induced neuropathic pain.

## Supplementary Materials

The following are available online at https://www.mdpi.com/2073-4409/9/2/320/s1, Table S1: Antibodies used in MELC and FACS analyses; Table S2: Internal standards and their concentration in the working solution for the LC-QTOFMS analysis; Table S3: Health status of tumor cell-injected mice; Figure S1: Body weight of tumor cell-injected mice; Figure S2: Tumor-induced downregulation of sphingomyelins in sciatic nerves; Figure S3: Tumor-induced downregulation of triglycerides and acylcarnitines in sciatic nerves; Figure S4: Proinflammatory macrophages and dendritic cells are in direct vicinity of epinerural adipocytes; Figure S5: Naïve sciatic nerves do not show immune cells at the sciatic nerve; Figure S6: Macrophage polarization in tumors and sciatic nerves.
