# Supplementary material for: Tumors Provoke Inflammation and Perineural Microlesions at Adjacent Peripheral Nerves
Source: Cells. 2020 Jan 29;9(2):320. doi: 10.3390/cells9020320 (PMC7072456; doi:10.3390/cells9020320)
Supplement: Supplementary file 1 [file cells-09-00320-s001.pdf]

Table S1:

Table S1: Antibodies used in MELC analyses.

Target	Company	Clone or order number
CD4	Southern Biotech	L3T4
CD8	BD Pharmingen	53-6.7
CD11b	BioRad	M1/70.15
CD11c	Miltenyi Biotec	N418
CD22	Miltenyi Biotec	130-102-576
CD31	BD Biosciences	MEC13.3
CD45	Miltenyi Biotec	30F11.1
CD86	Biolegend	GL-1
CD206	AbD Serotec	MCA22335FA
Ki67	eBioscience	SolA15
Ly6G	eBioscience	RB6-8C5
MHC II	Miltenyi Biotec	120-000-810
Siglec F	BD Pharmingen	E50-2440
TNFα	Miltenyi Biotec	130-109-720

Table S2:

Table S2: Internal standards and their concentration in the working solution for the LC-QTOFMS analysis.

Analyte	Concentration (ng/mL)
Arachidonic acid-d8	8.33
CE 18:1-d7	4650
Cer d18:1/16:0-d7	6.67
Cholesterol-d7	1330
DG 15:0/18:1-d7	157
LacCer d18:1/17:0	53.3
LPC 18:1-d7	317
LPC O-16:0-d4	16.7
LPE 18:1-d7	6.67
LPG 17:1	31.7
LPI 17:1	14.2
PC 15:0/18:1-d7	2110
PC O-18:0/18:1-d9	104
PE 15:0/18:1-d7	66.7
PE O-18:0/18:1-d9	0.933
PG 15:0/18:1-d7	63.3
PI 15:0/18:1-d7	113
PS 15:0/18:1-d7	261
SM d18:1/18:1-d9	393
TG 15:0/18:1-d7/15:0	759

Table S3:

Table S3: Scoring of the general health status at the latest recorded day.

Read out	E0771 d14	B16-F10 d10	MC57 d19
dehydrated	0	0	0
posture: bended	0	0	0
hunched	0	0	0
movements: reduced	0	0	0
paralysis	0	0	0
cramps	0	0	0
fur: piloerection	0	0	0
dirty	0	0	0
body openings:			
diarrea	0	0	0
handling: agressive	0	0	0
apathic	0	0	0
anxious	0	0	0
Others: skin rash	0	0	0
skin inflammation	0	0	0
injury	0	0	0

Scoring of the general health status was performed at a daily basis (n=16).
Score 0 depicts no change in comparison pretreatment conditions.

Figure S1:

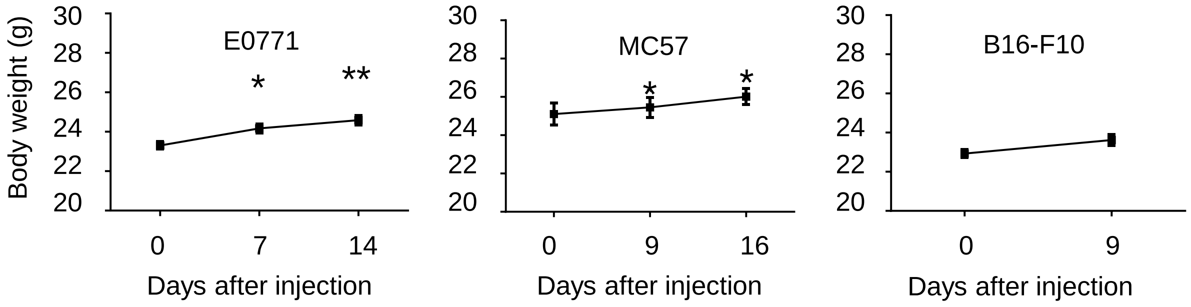

Figure S1: Body weight (g) of mice before and after tumor cell injection. Data are shown as mean ± S.E.M. (n=13).One-way ANOVA/Tukey's multiple comparison * $p<0.05$; ** $p< 0.01$

Figure S2:

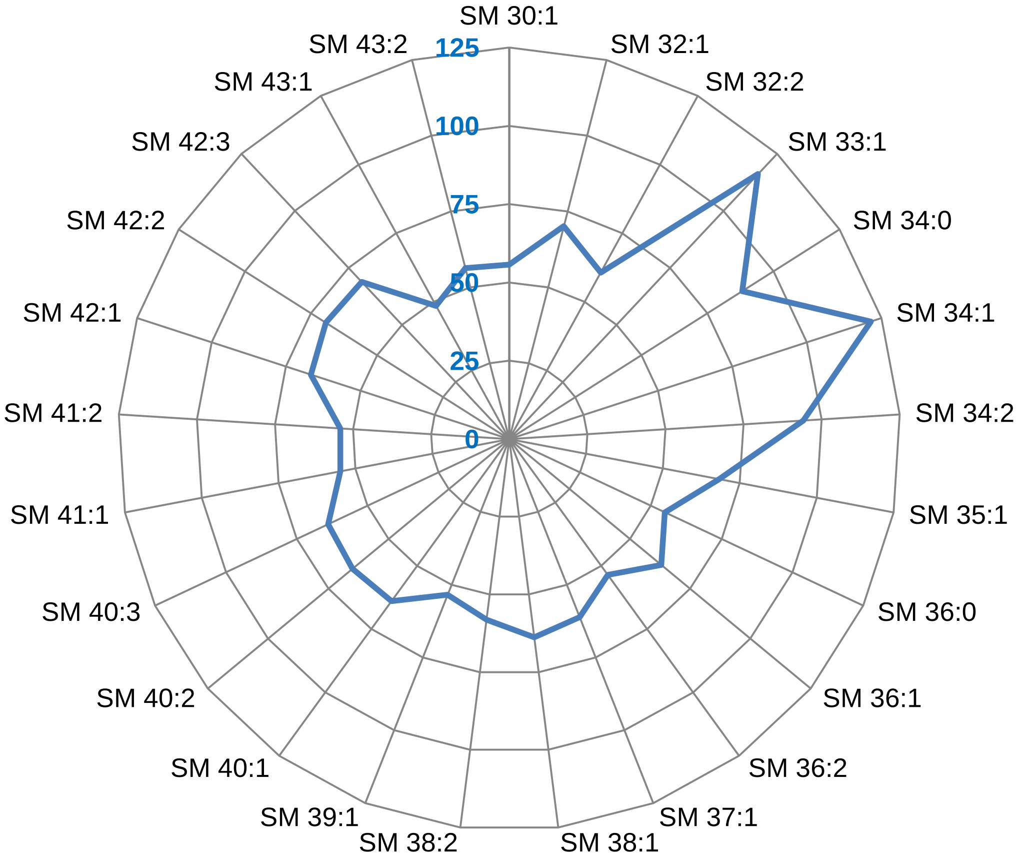

Figure S2: Tumor-induced downregulation of sphingomyelins in sciatic nerves. Lipidomic comparison of ipsi- and contralateral sciatic nerves 14 days after E0771 tumor cell injection. Shown are sphingomyelin concentrations. Data are mean (n=5) and are presented as level in the ipsilateral nerve in percent of the contralateral nerve (set as 100%).

Figure S3:

A

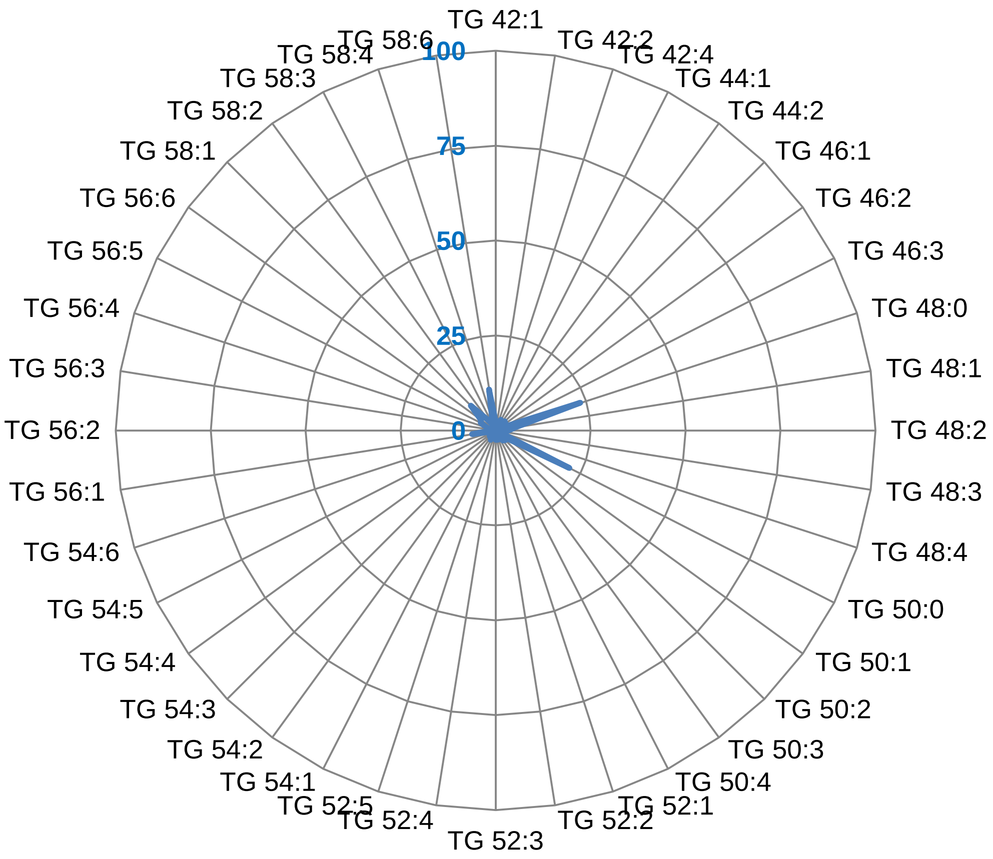

B

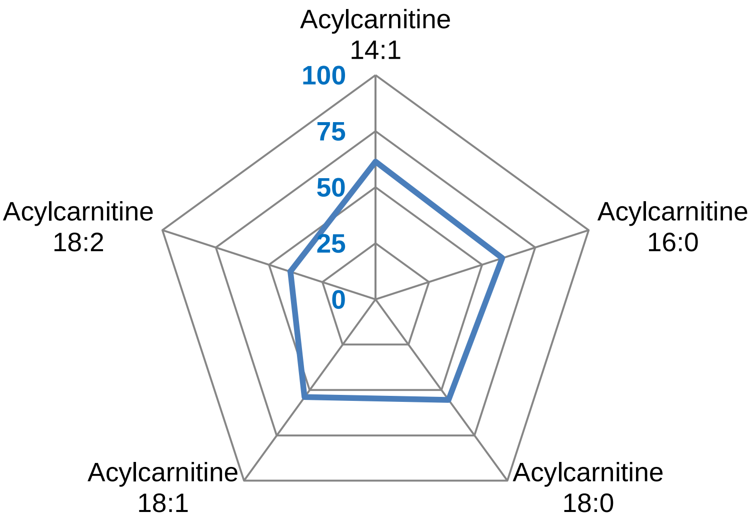

Figure S3: Tumor-induced downregulation of triglycerides and acylcarnitines in sciatic nerves. **(A,B)** Lipidomic comparison of ipsi- and contralateral sciatic nerves 14 days after E0771 tumor cell injection. Shown are triglycerides (panel A) and acylcarnitines (panel B). Data are mean (n=5) and are presented as level in the ipsilateral nerve in percent of the contralateral nerve (set as 100%).

Figure S4:

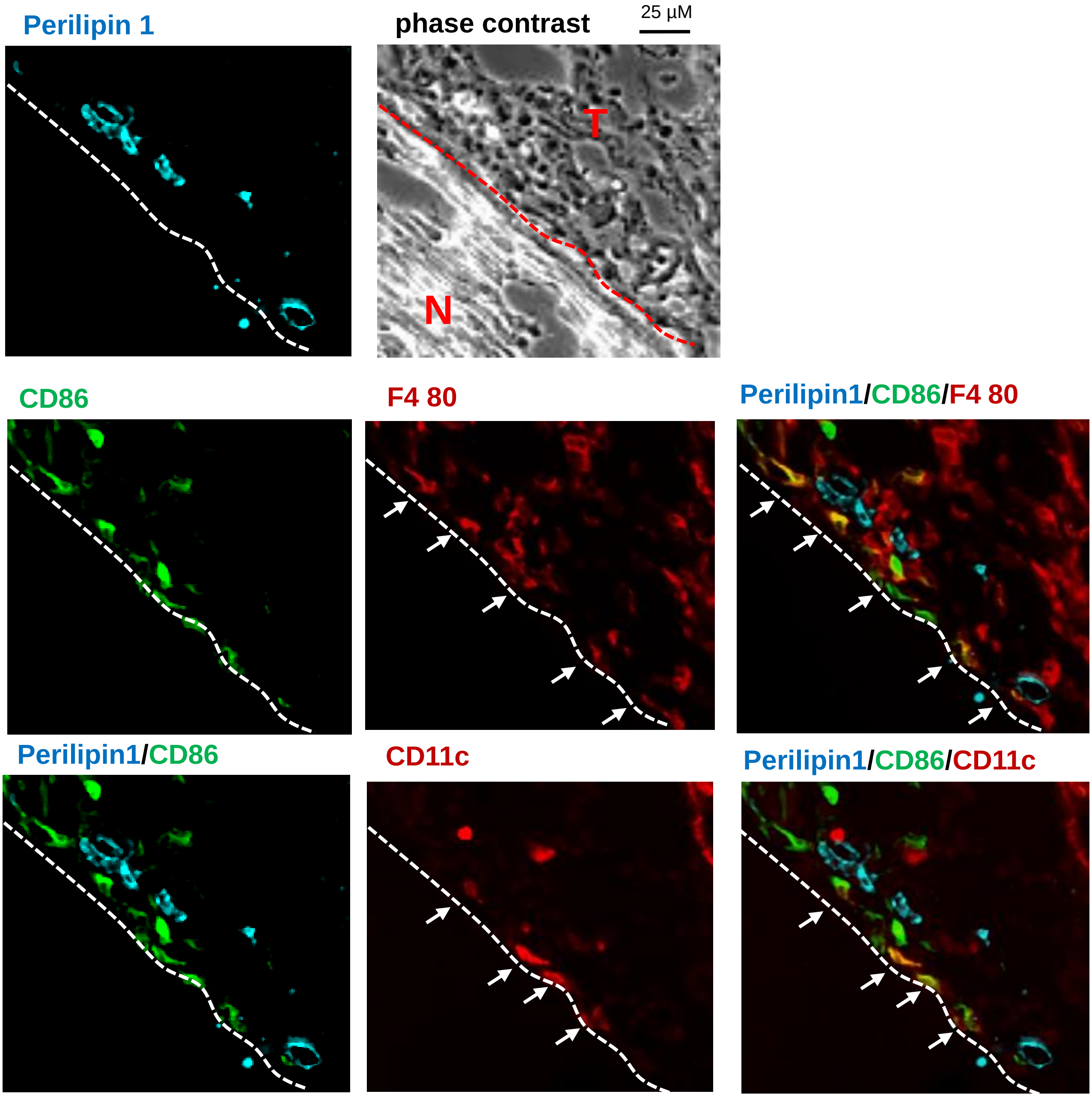

Figure S4: Proinflammatory macrophages (CD86+/F4 80+) and dendritic cells (CD86+/CD11c+) are in direct vicinity of epinerural adipocytes.
MELC staining of sciatic nerves from mice 14 days after injection with E0771 tumor cells. The white dotted lines depict the outer layer of the sciatic nerve. T, tumor tissue; N, sciatic nerve.

Figure S5:

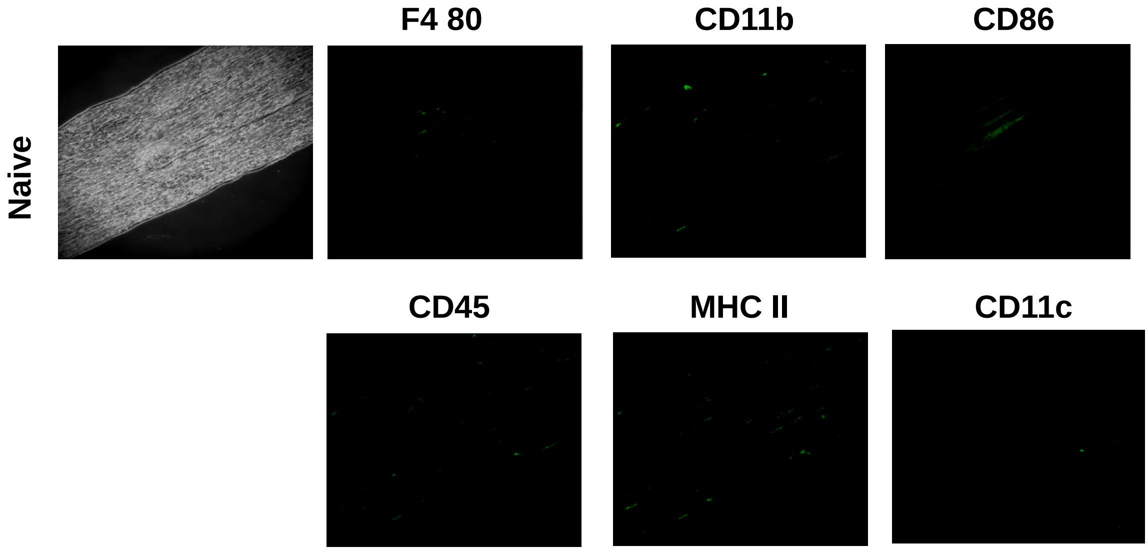

Figure S5: Macrophages or dendritic cells are not observed in sciatic nerves from naïve mice. MELC staining of sciatic nerves from naive mice show no immune cells within or attached to the sciatic nerve.

Figure S6:

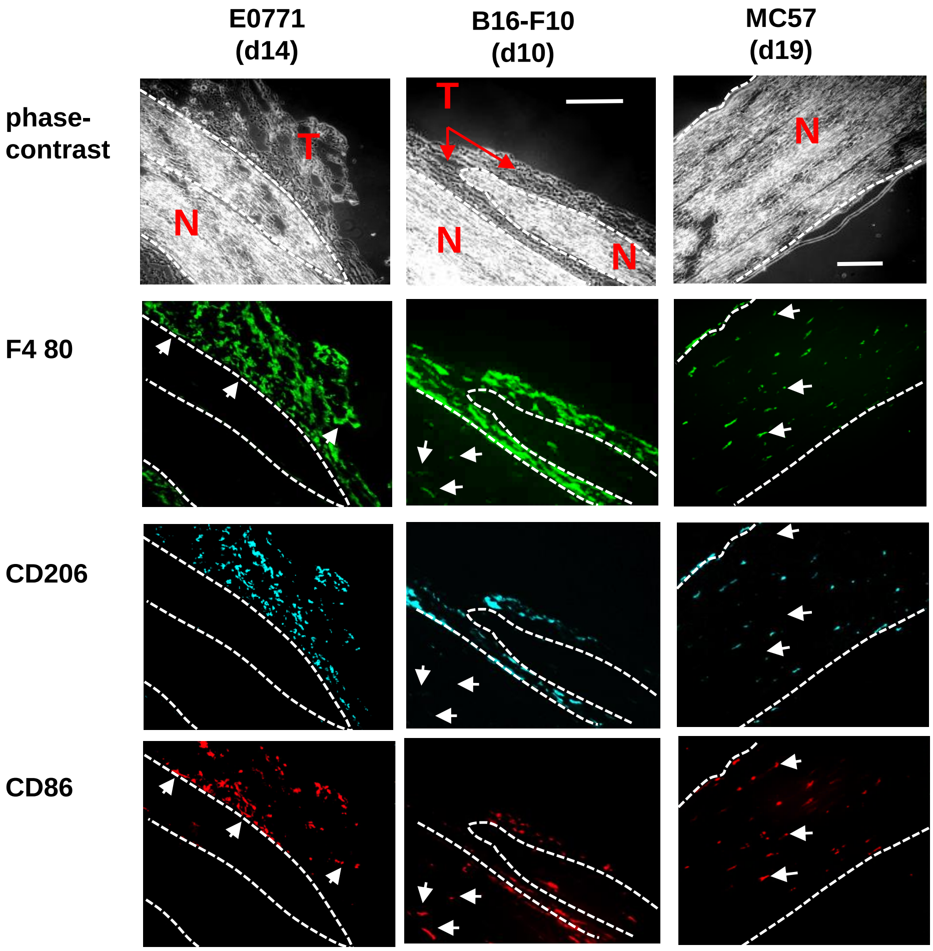

Figure S6: Macrophage polarization in tumors (T) and sciatic nerves (N).
MELC staining of sciatic nerves from mice after injection with E0771 (d14), B16-F10 (d10) or MC57 (d19) tumor cells (for E0771 see also Figure S4). Shown are stainings for F4 80 (macrophages), CD206 (M2 polarization) and CD86 (M1 polarization). MC57 tumors are not in contact with the sciatic nerve and are therefore not included in the images. White arrows depict examples for CD86-expressing macrophages inside the sciatic nerve. White dotted line depict the outlines of the sciatic nerves. White bars = 100 µm.
